# Regional Suppression of *Bactrocera* Fruit Flies (Diptera: Tephritidae) in the Pacific through Biological Control and Prospects for Future Introductions into Other Areas of the World

**DOI:** 10.3390/insects3030727

**Published:** 2012-08-10

**Authors:** Roger I. Vargas, Luc Leblanc, Ernest J. Harris, Nicholas C. Manoukis

**Affiliations:** 1 USDA-ARS, U.S. Pacific Basin Agricultural Research Center, 64 Nowelo St., Hilo, HI 96720, USA; E-Mails: ernest96822@lycos.com (E.J.H.); nicholas.manoukis@ars.usda.gov (N.C.M.); 2 Department of Plant Environmental Protection Sciences, University of Hawaii, Honolulu, HI 96822, USA; E-Mail: leblancl@ctahr.hawaii.edu

**Keywords:** parasitoids, Braconidae, Tephritidae, *Bactrocera*, Hawaii

## Abstract

*Bactrocera* fruit fly species are economically important throughout the Pacific. The USDA, ARS U.S. Pacific Basin Agricultural Research Center has been a world leader in promoting biological control of *Bactrocera* spp. that includes classical, augmentative, conservation and IPM approaches. In Hawaii, establishment of *Bactrocera cucurbitae* (Coquillett) in 1895 resulted in the introduction of the most successful parasitoid, *Psyttalia fletcheri* (Silvestri); similarly, establishment of *Bactrocera dorsalis* (Hendel) in 1945 resulted in the introduction of 32 natural enemies of which *Fopius arisanus* (Sonan), *Diachasmimorpha longicaudata* (Ashmead) and *Fopius vandenboschi* (Fullaway) were most successful. Hawaii has also been a source of parasitoids for fruit fly control throughout the Pacific region including Australia, Pacific Island Nations, Central and South America, not only for *Bactrocera* spp. but also for *Ceratitis* and *Anastrepha* spp. Most recently, in 2002, *F. arisanus* was introduced into French Polynesia where *B. dorsalis* had invaded in 1996. Establishment of *D. longicaudata* into the new world has been important to augmentative biological control releases against *Anastrepha* spp. With the rapid expansion of airline travel and global trade there has been an alarming spread of *Bactrocera* spp. into new areas of the world (*i.e.*, South America and Africa). Results of studies in Hawaii and French Polynesia, support parasitoid introductions into South America and Africa, where *B. carambolae* and *B. invadens*, respectively, have become established. In addition, *P. fletcheri* is a candidate for biological control of *B. cucurbitae* in Africa. We review past and more recent successes against *Bactrocera* spp. and related tephritids, and outline simple rearing and release methods to facilitate this goal.

## 1. Introduction

Fruit flies (Diptera: Tephritidae) are among the most economically important pests attacking soft fruits worldwide [1]. The *Bactrocera* genus is particularly important throughout the Pacific. It consists of at least 440 species distributed primarily in tropical Asia, Australia, and the South Pacific [1]. *Bactrocera* species are well-documented invaders and rank high on quarantine lists worldwide [2]. Polyphagy, superior mobility and dispersive powers, and high reproductive rates are among the common traits of invasive *Bactrocera* species. Throughout Pacific Island Nations, fruit flies have: (1) limited the development of a diversified tropical fruit and vegetable industry; (2) required that commercial fruits undergo quarantine treatment prior to export; and (3) provided a breeding reservoir for their introduction into other parts of the world due to unprecedented travel and trade between countries. Three invasive *Bactrocera* species (melon fly, *Bactrocera. cucurbitae* (Coquillett) (introduced in 1895) Figure 1b, oriental fruit fly, *Bactocera dorsalis* (Hendel) (1945) Figure 1a, and Malaysian fruit fly, *Bactrocera* *latifrons* (Hendel) (1983)), have been devastating to Hawaiian agriculture for over 100 years and have been studied extensively [3]. In addition to the development of area-wide technologies such as the sterile insect technique, protein bait sprays, and male annihilation, Hawaii has been a world leader in the development of classical, augmentative and conservation biological control approaches using parasitoid wasps (Hymenoptera: Braconidae) to suppress *Bactrocera* species.

**Figure 1:**
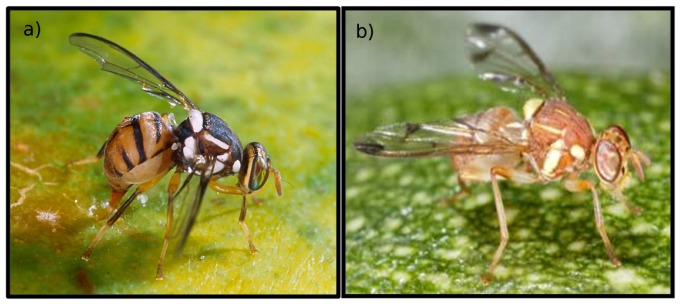
(**a**) Bactrocera dorsalis; (**b**) Bactrocera cucurbitae.

We anticipate that with the recent introductions of *B. cucurbitae*, *B. latifrons*, and *Bactrocera invadens* Drew, Tsuruta, and White into Africa and *Bactrocera carambolae* Drew and Hancock into South America [4,5], there will be increased interest in biological control. The objectives of this paper are to (1) review past and recent biological control work in Hawaii and French Polynesia, particularly with respect to the *Bactrocera* species; (2) summarize the various introductions throughout the region; (3) summarize successes and failures; (4) summarize rearing and release methods for future introductions; and (5) comment on prospects for future introductions into other parts of the world.

## 2. Biological Control of Fruit Flies in Hawaii

Introduction of invasive *Bactrocera* spp into Hawaii resulted in the initiation of extensive biological control programs. Initially these were classical biological control programs, but later, when mass rearing was perfected, augmentative releases were done. For example, establishment of *B. cucurbitae* in Hawaii in 1895 resulted in the introduction of eight species of hymenopterous parasitoids and six predators [6]. However, except for the parasitoid *Psyttalia fletcheri* (Silvestri), a widespread larval-pupal parasitoid of *B*. *cucurbitae* in India and introduced into Hawaii in 1916, the natural enemies were of little importance from the standpoint of biological control due to their scarcity and non-specificity to *B*. *cucurbitae* [7,8]. The host fruit species infested by *B*. *cucurbitae* appears to influence rate of parasitization by *P. fletcheri*. For example, Nishida [6] found little or no parasitization of larvae in papaya (*Carica papaya* L.), bell pepper (*Capsicum annuum* L.), or tomato (*Lycopersicon esculentum* Mill.), while Willard [7] reported that parasitization ranged from 7.3% to 29.8% in cucumber (*Cucumis sativus* L.) and was as high as 96.9% on wild bitter melon (*Momordica charantia* L.). Parasitization is also influenced by the location of host larvae within the plant. For example, in melons, where the larvae may be found in both vines and fruits, consistently higher parasitization was obtained in vines than in fruit [6]. Apparently, in vines, the larvae remain just beneath the epidermis throughout the developmental period, remaining within reach of the parasitoid’s ovipositor. In contrast, larvae infesting fruit have a tendency to burrow deeply into the fruit flesh during later stages of development and consequently become less accessible to parasitoids. Interestingly, *B*. *cucurbitae* is generally immune to the development of *Fopius arisanus* (Sonan), a very successful braconid parasitoid of *B*. *dorsalis* [9]. However, Harris *et al.* [9] demonstrated that concurrent releases of *F*. *arisanus* and *P*. *fletcheri* increased suppression of *B*. *cucurbitae* in patches of wild ivy gourd (*Coccinia grandis* L.) compared to releases of *P*. *fletcheri* alone. It is suspected that, although survival of *F*. *arisanus* in *B*. *cucurbitae* is low, mortality due to puncturing of eggs added to suppression. Nonetheless, *P*. *fletcheri* has remained the most important parasitoid of *B. cucurbitae* for almost a century [10].

With the introduction of *B. dorsalis* into Hawaii in 1945, the largest classical biological control program against fruit flies to date was undertaken to reduce its serious damage to fruits [8]. Thirty-two natural enemies were released between 1947 and 1952 [11]. *Diachasmimorpha longicaudata* (Ashmead) increased rapidly following its release in 1948, but suddenly lost its dominant position during the latter half of 1949 to *Fopius vandenboschi* (Fullaway), which was later superseded by the egg-pupal parasitoid *F. arisanus* [12,13,14]. Since its establishment, *F. arisanus* has resulted in a dramatic reduction in fruit infestation in Hawaii through a high level of *B. dorsalis* parasitism (65%–70%), and has remained the dominant parasitoid species [15,16]. Clearly, given the success of fruit fly biological control in Hawaii, a wealth of information was generated that can apply to other areas throughout the Pacific and the world.

Although no parasitoids were deliberately introduced to control *B*. *latifrons*, the third economically important *Bactrocera* species detected in Hawaii in 1983, five primary parasitoid species have been recovered from individually held *B. latifrons* puparia: *F*. *arisanus*, *Psyttalia incisi* (Silvestri), *D*. *longicaudata*, *Diachasmimorpha tryoni* (Cameron) and *Tetrastichus giffardianus* Silvestri [17]. Of these, *F. arisanus* was the predominant species recovered at study sites [17].

## 3. Biological Control Programs for Fruit Flies in French Polynesia

The invasion of *B. dorsalis* (1996) was the most devastating of four accidental introductions of economically important *Bactrocera* species into French Polynesia, the others being *Bactrocera kirki* (Froggatt) (1928), *Bactrocera tryoni* (Froggatt), Queensland fruit fly, (1970), and *Bactrocera xanthodes* (Broun), Pacific fruit fly, (1998) [18]. *Bactrocera dorsalis* has been reported in the Society, Austral and Marquesas Islands while *B. xanthodes* is confined to the Austral Islands. Studies in French Polynesia are unique in that emergence data from large numbers of fruit samples were compared before and after releases of *F. arisanus* on Tahiti Island over ca. a 10 year period [19,20]. Starting in 2002, 10 parasitoid shipments from Hawaii, over 500,000 insects, were dispersed in two major locations and 10 minor locations around Tahiti Island. *Fopius arisanus* was established throughout all 21 communities within 3 years on Tahiti Island and nearby Moorea Island [19]. It became so abundant in guava fruits that parasitoids recovered from wild fruits were used to establish it on the Society Islands of Huahine, Tahaa, and Raiatea where *B. dorsalis* had spread [19]. By 2009 mean (±SD) parasitism of fruit flies infesting *P. guajava*, *Inocarpus fagifer* (Parkinson) Fosberg (Polynesian chestnut) and *Terminalia catappa*. (tropical almond) fruits on Tahiti Island was 64.8 ± 2.0% [20]. A second parasitoid, *D. longicaudata*, was released in 2007 [20]. Five shipments of *D. longicaudata* (of approximately 5,000 each with a total of about 10,000 surviving wasps) were made between September 2007 and August 2008. Although also becoming widespread, parasitism rates of *D. longicaudata* have not been higher than 10% [20]. As a result of parasitoid introduction, numbers of *B. dorsalis*, *B. tryoni* and *B. kirki* emerging (per kg of fruit) declined sharply. For example, for *P. guajava* there was a decline, between 2003 and 2009, of 92.3, 96.8, and 99.6% for each of the fly species, respectively [19]. Analysis of co‑infestation patterns (1998–2009) of *B. dorsalis*, *B*. *tryoni*, and *B*. *kirki* in four main host fruits suggest that *B. dorsalis* has become the most abundant species wherever it occurs [20]. Establishment of *F*. *arisanus* in French Polynesia is the most successful example of classical biological control of fruit flies in the Pacific outside of Hawaii and it was secondarily introduced from Tahiti as *B. dorsalis* spread to other French Polynesian islands, most recently to the Marquesas Islands (Nuku Hiva, Hiva Oa and Fatu Hiva) [20].

French Polynesia is comprised of over 118 islands and atolls scattered over approximately 2,500,000 km^2^ of ocean. *Bactrocera dorsalis* is currently established in the Society, Marquesas and Austral Islands of French Polynesia. Initially it was envisioned that *F. arisanus* could be mass reared in Hawaii at an estimated cost of US $2,000 per 1,000,000 parasitoids [21], and transferred to other islands as *B*. *dorsalis* was progressively spreading throughout French Polynesia. However, when *F. arisanus* became numerous in fruits infested with *B*. *dorsalis* on Tahiti Island, it became more cost effective to recover wasps from field-collected fruits and ship them to the outer islands, than to mass rear them in the laboratory. This is now the preferred approach for shipments and quick establishment to new islands where *B*. *dorsalis* has spread [19].

## 4. Hawaii as a Source of Parasitoids throughout the Pacific

**Figure 2:**
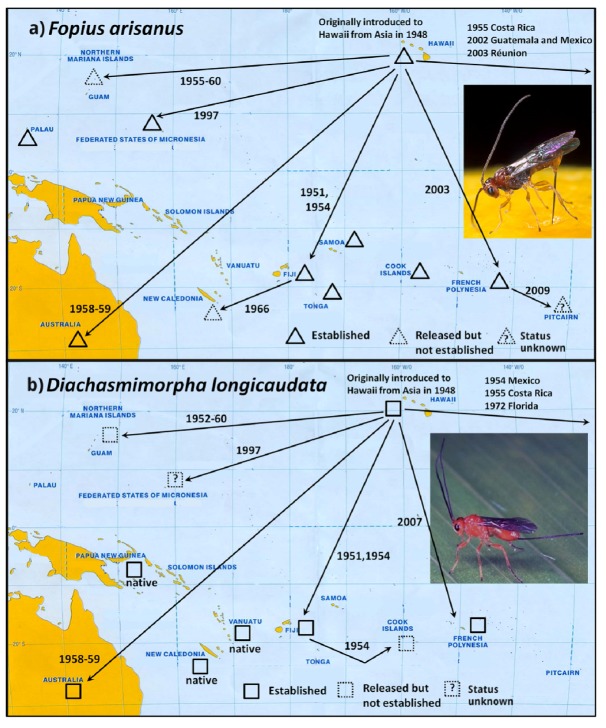
Introductions of parasitoids (Braconidae) for fruit fly biological control in the Pacific. (**a**) *Fopius arisanus*; (**b**) *Diachasmimorpha longicaudata.*

From 1935 to 2008 Hawaii has been very active in exporting fruit fly parasitoids throughout the world for suppression of a variety of fruit fly species with varying degrees of success [20,22]. Originally established in Hawaii around 1948, *F. arisanus* has since been released in 11 Pacific Island countries against various *Bactrocera* spp Figure 2a. Establishment has been confirmed in seven countries (Table 1). Similarly, *D*. *longicaudata*, also introduced into Hawaii in 1948, has been released in six countries with establishment confirmed in three countries (Figure 2b, Table 1). Throughout the Pacific region parasitism rates have varied depending on parasitoid species (*F. arisanus* or *D. longicaudata*), target host fruit fly species, and host fruit (Table 1). The highest *F. arisanus* parasitism rates have been obtained with the *B*. *dorsalis* complex species in Hawaii, French Polynesia, and Palau. *Psyttalia fletcheri* was established in Hawaii in 1916 and has been released and established in the Northern Mariana Islands (starting in 1950) and the Solomon Islands (in 1997) [23].

The extensive biological control programs in Hawaii have also resulted in shipments of parasitoids to various localities in the US and Latin America [22], with the largest programs in Mexico, Costa Rica and Florida. The program in Costa Rica was in direct response to the establishment of *Ceratitis capitata* (Wiedemann), Mediterranean fruit fly, and its subsequent expansion to the rest of Central America [22]. Costa Rica then became the source of parasitoid introductions to 11 other American countries. Most of the control efforts targeted *C*. *capitata* and *Anastrepha* spp. in Central America (Nicaragua, Panama, El Salvador, Guatemala and Trinidad) and *C*. *capitata* and *Anastrepha fraterculus* (Wiedemann), South American fruit fly, in South America (Argentina, Bolivia, Peru, and Venezuela) [22]. The two most successful introduced parasitoids were *D*. *longicaudata* and the eulophid *Aceratoneuromyia indica* (Silvestri), both larval parasitoids [22]. These have become established on *A*. *fraterculus*, *Anastrepha ludens* (Loew) (Mexican fruit fly), *Anastrepha obliqua* (Macquart) (West Indian fruit fly), *Anastrepha serpentina* (Wiedemann) (sapote fruit fly), and *Anastrepha striata* Schiner (guava fruit fly) [22]. In Florida, populations of *Anastrepha suspensa* (Loew) (Caribbean fruit fly) decreased by 40% in the years following releases of the parasitoids *Doryctobracon areolatus* (Szepligeti) and *D. longicaudata* [24].

**Table 1:** Percent parasitism by *F. arisanus* and *D. longicaudata* in various countries where they have been introduced.

Country	Parasitoids	Target economic species	Assessment period	*Artocarpus* *altilis*	*Averrhoa* *carambola*	*Carica* *papaya*	*Citrus* spp ^1^	*Inocarpus* *fagifer*	*Psidium* *cattleianum*	*Psidium* *guajava*	*Syzygium* spp	*Terminalia* *catappa*	Reference ^2^
Australia ^3^	*F.* *arisanus*	*B.* *tryoni*	1960–64	---	0–78%	---	0–21%	---	0–2%	0–21%	---	---	[25,26]
Australia ^4^	*D.* *longicaudata*	*B.* *tryoni*	1963–65	---	---	---	---	---	1%–5%	0–7%	---	---	[26]
Cook Islands	*F.* *arisanus*	*B.* *melanotus*, *B. xanthodes*	1991–92	0.6%	---	4.6%	---	1.0%	---	11.5%	5.4%	10.6%	RFFP
Fiji Islands	*F.* *arisanus*	*B.* *passiflorae*, *B. xanthodes*	1959–63	---	---	---	21.4%	0.5%	54.8%	22.1%	---	---	[27,28]
Fiji Islands	*D.* *longicaudata*	*B.* *passiflorae*, *B. xanthodes*	1959–63	---	---	---	0.3%	8.0%	6.5%	2.1%	---	---	[27,28]
Fiji Islands	*F.* *arisanus*	*B.* *passiflorae*, *B. xanthodes*	1990–99	3.3%	---	---	23.3%	30.3%	---	23.8%	6.2%	2.3%	RFFP
French Polynesia ^5^	*F.* *arisanus*	*B.* *dorsalis*	2005–09	34.7%	38.5%	32.4%	25.9%	50.2%	53.4%	54.2%	58.7%	45.5%	RFFP
French Polynesia ^5^	*D.* *longicaudata*	*B.* *dorsalis*	2008–09	3.5%	0.0%	0.8%	1.3%	2.4%	8.8%	0.6%	---	2.2%	[20]
Hawaii (Kauai)	*F.* *arisanus*	*B.* *dorsalis*	1988–89	---	---	---	35.2%	---	59.8%	58.6%	50.4%	---	[16]
Hawaii (Kauai)	*D.* *longicaudata*	*B.* *dorsalis*	1988–89	---	---	---	0.3%	---	2.6%	0.2%	0.8%	---	[16]
Palau	*F.* *arisanus*	*B.* *philippinensis*, *B. frauenfeldi*	2001	---	22.2%	---	---	---	---	4.5%	11.7%	---	RFFP
Samoa	*F.* *arisanus*	*B.* *kirki*, *B. xanthodes*	1991–95	---	---	0.8%	---	---	---	6.8%	---	0.4%	RFFP
Tonga	*F.* *arisanus*	*B.* *facialis*, *B. kirki*, *B. xanthodes*	1991–95	0.7%	---	---	---	0.1%	---	1.4%	1.2%	1.8%	RFFP

^1^ *C. paradisi*, *C. reticulata* and *C. sinensis* in Australia; *C. latifolia*, *C. maxima* and *C. sinensis* in French Polynesia; *C. maxima* and *C. sinensis* in Fiji, and *C. sinensis* in Hawaii; ^2^ Regional Fruit Fly Project (RFFP): Intensive host surveys carried out under the Regional Fruit Fly Projects in the Pacific; ^3^ Northern Queensland (Cairns area); ^4^ Lord Howe Island; ^5^ Tahiti Island.

## 5. Rearing and Release Protocol

We focus here on *F. arisanus* as a useful example of procedures for rearing fruit fly parasitoids, because it has historically been considered difficult to rear and maintain under colony conditions [13,14,22,29,30,31]. Complications with establishing and maintaining *F. arisanus* in the insectary might arise from the fact that it is an ovo-parasitoid, one of only three known species of opiine parasitoids to infest the host during that stage [32]. Despite these difficulties, United States Department of Agriculture-Agricultural Research Service (USDA-ARS) researchers were able to develop a robust protocol for establishing and maintaining *F. arisanus* in colony [33,34,35]. This basic protocol was recently described in a video-article [36], in an effort to facilitate the adaption of these methods in other parts of the world. We provide an overview here of the rearing method; interested readers are referred to the sources above for further details.

**Figure 3:**
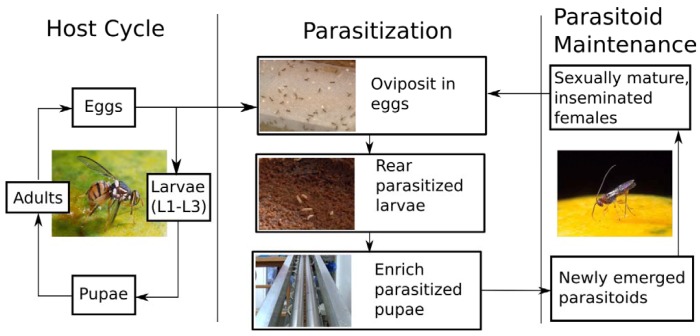
Rearing protocol for *Fopius arisanus* and related parasitoids.

The current protocol used at USDA-ARS in Hilo, HI involves three major components: (1) maintaining a stock of host fruit flies (*B. dorsalis* is mainly used for this purpose, although *F. arisanus* is known to parasitize other species); (2) parasitization of the host eggs, development of the parasitoid in the host and enrichment of the proportion of parasitoids; and (3) maintenance of the parasitoid colony under conditions that allow high survivorship, sexual maturation and insemination of females. These three components are shown schematically in the flow chart in Figure 3. The first component, maintaining host fruit fly stocks, has been extensively covered by Vargas [37] and will not be discussed here.

Rearing of *F*. *arisanus* has been covered in detail by Bautista *et al.* [35] and most recently in a video by Manoukis *et al.* [36] and will only be summarized here. The second component, “Parasitization”, is the most technically involved and specific to *F. arisanus*. Fruit fly eggs are placed on an agar or glycerin substrate that has been prepared in a shallow dish. These dishes are then placed under the screened bottom of small holding cages containing mature *F. arisanus*. Females are then given 21 h to parasitize the eggs. The substrate with eggs is then placed on a dish of larval diet within a large covered fiberglass pupation container, and kept in the dark at 27 °C and 80% RH for one week. Pupae are then sifted from vermiculite or sand at the bottom of the fiberglass container and sorted by size. Pupae that are between 0.165 and 0.226 cm in diameter contain mostly parasitoids [35]; these are the pupae that are held for emergence after 7 more days.

For the third component, Parasitiod Maintenance, there are a few important practices worthy of mention. *Fopius arisanus* should be kept in small cages (approximately 25 cm^3^). These should have a removable glass front, screened sides, top and bottom, and an access port in the back. The glass front should be tinted along the bottom edge (approx. 6 cm) to prevent parasitoids from accumulating in the corners and crowding each other. Parasitized pupae should be placed in a container with a coarse screened lid that allows *F. arisanus* to pass through while trapping any remaining fruit flies.

*Fopius* *arisanus* cultures are held in a room maintained at 24 °C and 45% RH with a 12:12 photoperiod and good ventilation. Feeding is accomplished by streaking spun honey along the top of the holding cages at least three times per week. Agar blocks are also placed on top of the holding cages to provide moisture for the parasitoids.

**Figure 4:**
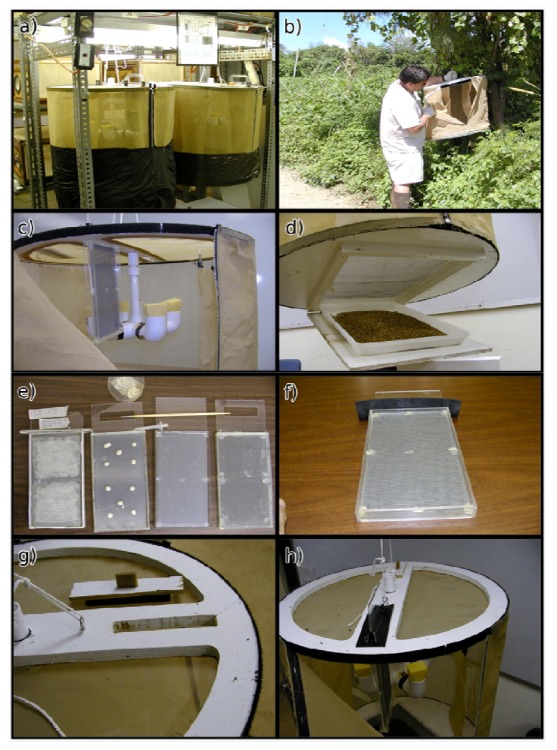
(**a** to **h**) The cylindrical cage for mass-rearing and release of *Fopius arisanus*.

As an alternative to small cubical cages, a cylindrical cage (Figure 4) was also developed for the dual purpose of rearing (Figure 4a) and transport and release (Figure 4b) of the parasitoids [9]. The 60 × 65 cm screened cage can hold 10,000 adult *F. arisanus*. Some larger “release-only” cages used in the Hawaii Area-Wide Pest Management (AWPM) program, hold up to 30,000 parasitoids [38]. The cage shown in the figure includes a water dispenser and a vertical stinging unit (Figure 4c), and food is provided as spun honey smeared on the outside walls of the cage [9]. To initiate a colony, parasitized pupae are placed in a tray in a trapdoor below the cage (Figure 4d) [9]. Emerging parasitoids escape through the screened cage floor, while flies are excluded because of small screen mesh size (0.1 cm^2^) [35]. *Bactrocera dorsalis* eggs as host material are provided on a stinging unit (Figure 4e) that consists of agar poured over a rigid screen mesh, covered with tissue paper, over which eggs are evenly distributed using a fine paintbrush, and covered with a fine fabric screen to hold eggs in place [35]. The prepared unit (Figure 4f) is inserted through a slot on the cage roof (Figure 4g), allowing wasps to sting the fly eggs held in the vertical unit (Figure 4h) [35]. After 24 h of exposure, the unit is removed and the agar plate with parasitized eggs is placed over larval diet [35].

## 6. Prospects for the Future

*Fopius* *arisanus* occurs from south India to Taiwan, and has been introduced and established in Australia, Cook Islands, Costa Rica, Fiji, Hawaii, Mauritius, Samoa, Tonga, Reunion, and Israel [39,40]. *Diachasmimorpha longicaudata* occurs throughout southeast Asia, east to Papua New Guinea, and has been introduced and established in Australia, Fiji, Mexico, Costa Rica, Florida and Trinidad on a variety of hosts [40], and was more recently recorded from Vanuatu [39]. *Psyttalia fletcheri* is a widespread larval-pupal parasitoid of *B*. *cucurbitae* and occurs throughout India, Sri Lanka, Malaysia and Indonesia, and has been established in Hawaii, the Solomon Islands and Northern Marianas [40]. Perhaps no fruit fly parasitoids have been studied more thoroughly than *F. arisanus*, *D*. *longicaudata* and *Psyttalia concolor* (Szépligeti). Because of its habit of attacking host eggs, which are more exposed below the fruit skin surface than larvae, *F. arisanus* can achieve high levels of parasitism, often surpassing 50% in the field [16,41]. In Hawaii, its introduction resulted in a 95% reduction in the *B*. *dorsalis* population, compared to the 1947–1949 peak abundance of *B*. *dorsalis* [42]. Furthermore, *F*. *arisanus* also became the major parasitoid of *C*. *capitata* in Hawaii [42,43]. Haramoto and Bess [15] reported that the mean number of fruit fly pupae (*B*. *dorsalis* and *C*. *capitata*) collected from *Coffea arabica* L. (coffee) berries in Kona, Hawaii, decreased from 23.6 pupae per 100 fruits (8.7% parasitism) in 1949 to 5.2 (66.6% parasitism) in 1969. With this level of impact on infestation level, establishment of *F. arisanus* has reduced the threat of movement of fruit flies to the mainland from Hawaii. When *D. longicaudata* was established in French Polynesia to complement *F. arisanus*, data suggested it rarely accounted for more than 8.8% parasitism, but still has become widespread [20]. Nonetheless, establishment of *D. longicaudata* has increased total parasitoid mortality and more time may be necessary for *D. longicaudata* populations to increase [20]. During surveys, one specimen of *Diachasmimorpha tryoni* (Cameron) was reared from *Inocarpus fagifer* (Polynesian chestnut) on Tahiti in October 2003, probably a result of previous releases against *B. kirki* previously not known to have established [44]. In Florida, populations of *A*. *suspensa* decreased by 40% in the years following releases of the parasitoids *Doryctobracon areolatus* (Szépligeti) and *D*. *longicaudata* [24]. The larval parasitoid, *Psyttalia* cf. *concolor*, collected from tephritids infesting coffee in Kenya and reared on *C*. *capitata* by USDA-Animal Plant Health Inspection Service, Plant Protection and Quarantine in Guatemala, was recently imported into California for biological control of olive fruit fly, *Bactrocera oleae* (Gmelin) (introduced in 1998). Further details are found in Yokoyama *et al.* [45].

*Fopius* *arisanus* polyphagy has been studied extensively [46]. For example, in Hawaii, it attacks eggs of *B. dorsalis*, *C. capitata*, and *B. cucurbitae*, but does not develop successfully in *B. cucurbitae* [13,47]. Sometimes *F. arisanus* adults emerge from field-collected fruits infested by several fruit fly species, so exact host relationships cannot be inferred accurately, unless the fruit fly species can be distinguished at the pupal stage [25]. Vargas *et al.* [43] segregated *B. dorsalis* and *C. capitata* pupae from field collections and found *F. arisanus* to also be the dominant *C. capitata* parasitoid in Hawaii. In Australia, Quimio and Walter [46] were able to rear *F. arisanus* on *B. tryoni* in the laboratory and it has also been recovered from the field [25], but percent parasitism is lower than on *B. dorsalis* (Table 1). Although *B*. *tryoni* parasitism in French Polynesia was never confirmed due to mixed infestation, *F. arisanus* was reared in the laboratory on *B. tryoni* [38]. It is suspected, as was the case in Hawaii with *C. capitata*, that *F. arisanus* also has an impact on lesser preferred species, such as *B. tryoni*, by increasing its numbers on a large *B. dorsalis* population. Furthermore, parasitism of *B. tryoni* and *B. kirki* eggs in fruits with mixed infestations may result in significant mortality of host species in the egg or larval stage, although parasitoids may have lower survivorship, as has been shown with *B. cucurbitae* [9,48].

## 7. Conclusions

In surveys in Hawaii, the egg-pupal parasitoid *F. arisanus* and the larval-pupal parasitoid *D. longicaudata* constitute 87.5%–95.1% and 0.9%–9% of the parasitoid guild, respectively, and are very common in tree fruits, particularly *P. guajava* and *P. cattleianum* (Sabine), strawberry guava [16]. Since its establishment in Hawaii, *F. arisanus* has resulted in a dramatic reduction in infestation of fruit through a high level of *B. dorsalis* parasitism (65%–70%) [15]. In Tahiti, parasitism on *P.* *guajava*, *I. fagifer*, and *T.* *catappa* fruit collections has increased from approximately 50% to 65% from 2006 to 2009 [20]. These percentages are very similar to those obtained in Hawaii and the observed increases during the period of *D.* *longicaudata* establishment would suggest few negative effects of *D. longicaudata* on *F. arisanus* [20].

The impact of *F*. *arisanus* releases has not always been as impressive in locations outside of Hawaii and French Polynesia to date [4]. For example, it was released and recovered in Costa Rica, but its impact has not been high, although little information is available on its present status or distribution on coffee farms, where *C. capitata* infests fruits [49]. Similarly, in Australia, *F*. *arisanus* was introduced from Hawaii and was established on the native *B*. *tryoni* in 1962, but reputedly had only a negligible effect [46]. Likewise, parasitism has been lower on the species of *Bactrocera* endemic to south Pacific Islands (Table 1) than on the *B. dorsalis* complex species.

The role of parasitoids were tested in the Hawaii AWPM fruit fly program at three levels of application: (1) conservation; (2) augmentative releases; and (3) classical releases [3]. *Fopius arisanus* and *P*. *fletcheri* were reared and released in wild guava and cucurbit patches, respectively, near agro-ecosystems [3], with the objective of demonstrating a cost-effective, sustainable technology that could be integrated with reduced risk bait sprays (*i.e.*, GF-120 Fruit Fly Bait) and male annihilation treatments [Specialized Pheromone Lure Application Technology-Male Annihilation Technique (SPLAT-MAT)-Methyl Eugenol-Spinosad]. In augmentative releases of *P. fletcheri* against melon fly, numbers of *B*. *cucurbitae* emerging from fruits placed inside field treatment cages were reduced by up to 21 fold and numbers of parasitoids were increased by 11 fold [10]. In open field releases of *P*. *fletcheri* into ivy gourd patches throughout the Kailua-Kona area, parasitism rates were 4.7 times higher in release plots compared to those in control plots. However there was no significant reduction in emergence of flies from fruits. Similarly, in releases of *P*. *fletcheri* in zucchini plots in Waimea, there was an increase in parasitoid recovery rates, but no reduction in melon fly damage. *Fopius arisanus* was also tested as an augmentative tool in small plots of guava in Waimea where the existing population of *F*. *arisanus* was low. Levels of parasitism were increased, but infestation was not reduced [3]. Therefore although augmentative releases of parasitoids were shown to increase parasitism in the field, limited rearing capacity, the high cost and the limited impact at reducing fruit fly infestations below economic thresholds limited their level of implementation in a sustainable AWPM-program. On the other hand, classical biological control was demonstrated to be very cost effective and sustainable in the French Polynesia program.

The establishment of natural enemies of invasive tephritid fly pests may have significant and beneficial impacts in regions otherwise lacking in natural enemies. Results from Hawaii support introduction of *F*. *arisanus* and *D*. *longicaudata* into South America and Africa, where *B. carambolae* and *B. invadens* have become established, respectively [4,5]. It is also thought that *F. arisanus* and *D*. *longicaudata* evolved in areas where the *B*. *dorsalis* complex species are indigenous and their success is likely to be higher on species of that complex than other fruit flies. Given *F. arisanus* attacks the egg stage and forages on ripening fruits, while *D*. *longicaudata* attacks third instar larvae and tends to forage among fallen fruits [38], they are, to an extent, complementary to each other. Furthermore, based on data collected in Hawaii, *F*. *arisanus* and *D*. *longicaudata* could also be released against *B*. *latifrons*, which has recently invaded the African continent, and the peach fruit fly, *B. zonata* (Saunders), in Africa and the Indian Ocean region (e.g., [50]). *Pysttalia fletcheri* is also a candidate for releases in Africa where *B*. *cucurbitae* has invaded.
